# Balanced fluid bolus: Should we prefer balanced crystalloids over “normal” saline?

**DOI:** 10.1038/s41372-026-02622-z

**Published:** 2026-03-13

**Authors:** Kelsey Carrigan, Satyan Lakshminrusimha

**Affiliations:** Department of Pediatrics, Division of Neonatology, UC Davis Children’s Hospital, Sacramento, CA USA

**Keywords:** Paediatrics, Adverse effects

## Abstract

A fluid bolus is administered to correct hypoperfusion, hypotension, and metabolic acidosis for resuscitation at birth or in the neonatal intensive care unit (NICU). However, there is a lack of evidence-based support for any particular fluid choice. The most used intravenous fluid is 0.9% sodium chloride or “normal saline” (NS). There is a growing body of evidence that NS is associated with hyperchloremic metabolic acidosis and acute kidney injury. Balanced crystalloid solutions such as lactated Ringer’s solution and solutions with a more physiological composition similar to plasma may offer some advantages compared to NS based on adult literature. Here we discuss indications and challenges with the use of balanced crystalloids and propose a practical approach to fluid choice in the NICU. Further research is needed to better understand indications for and optimal fluid therapy to correct hypoperfusion and metabolic acidosis in neonates.

## Introduction

Crystalloids are commonly administered for fluid resuscitation, yet there remains a lack of evidence-based support for the most effective and safest intravenous (IV) fluid bolus for preterm and term neonates in the delivery room and neonatal intensive care unit (NICU) [1]. Historically, 0.9% sodium chloride (commonly referred to as “normal saline”, NS) has been the most frequently used resuscitation fluid, but several studies have linked it with hyperchloremic metabolic acidosis, acute kidney injury, and death [2]. Alternatively, balanced crystalloid solutions, such as lactated Ringer’s (LR), Plasma-Lyte [Baxter Healthcare Corporation, Deerfield, IL, USA] (PL) and Normosol-R [Otsuka ICU Medical, Austin, TX, USA] (NoR), are designed to mimic the composition of human plasma. In the 1880’s, the original Ringer’s solution was formulated when Sydney Ringer incidentally observed that addition of inorganic salts to distilled water preserved the rhythmicity of frog heart muscle ex vivo better than distilled water alone [3, 4]. Ringer’s original formula was further modified by pediatrician Alexis Hartmann with an intention to correct metabolic acidosis in sick children by adding sodium lactate [5] making the LR solution in use today.

Metabolic acidosis is common in preterm and term newborns and is associated with various conditions such as hypotension, hypoperfusion, asphyxia, sepsis, and persistent pulmonary hypertension, and can increase mortality. In critically ill infants, severe tissue-level hypoxia, secondary to either poor perfusion (ischemia) or oxygen delivery (hypoxia), can shift metabolism towards anaerobic glycolysis with the generation of hydrogen ions and lower energy production. Base therapy, such as acetate, bicarbonate, citrate, and lactate, is commonly used in the NICU and may help correct metabolic acidosis [6].

## Physiologic basis for IV fluid bolus therapy in neonates

IV fluid boluses in neonates aims to restore circulating blood volume and cardiac preload to improve tissue perfusion and oxygen delivery during cardiovascular compromise. Fluid bolus therapy assumes that preload may be low and an increase in intravascular volume may optimize cardiac output based on the infant’s position on the Starling’s curve [7]. However, in the absence of overt blood or fluid loss, it is tough to predict the need for a fluid bolus. A common bolus regimen is 10 mL/kg infused over a median duration of 30 min [8], yet evidence for its effectiveness is limited. An increase in extracellular fluid volume before postnatal diuresis has been associated with unfavorable outcomes, particularly in preterm infants [9]. Additionally, clinicians have reported minimal or no clinical improvement in mean blood pressure in 40% of cases where IV fluid bolus therapy was used to treat hypotension [8]. In preterm neonates, the volume kinetics and distribution of crystalloids may depend on infusion rate, physiological state, and vascular permeability and tone [10, 11]. Extremely preterm infants face profound challenges in maintaining fluid and electrolyte homeostasis due to incomplete nephrogenesis, functional renal immaturity, and an immature skin barrier [12]. These vulnerabilities directly impact fluid management decisions and require careful consideration of both the type and volume of fluids administered.

## Comparing crystalloids

Despite being commonly referred to as ‘normal’, NS is neither physiologically normal as it contains 154 mM/L of both sodium and chloride nor biochemically a one normal (1 N) solution (which would be 58.5 g of NaCl in 1 L of water). Balanced crystalloid solutions have electrolyte compositions that better approximate human plasma and have added buffering agents, such as lactate, acetate, and gluconate, to generate bicarbonate and neutralize excess hydrogen ions (Table 1).

**Table 1:** Composition of common IV solution types and human plasma [34, 35].

		NS	LR	PL	NoR	Plasma
Electrolytes (mM/L)	Sodium	154	130	140	140	136-145
	Chloride	154	109	98	98	98-106
	Potassium	-	4.0	5.0	5.0	3.5-5.0
	Calcium	-	2.7-3.0	-	-	2.2-2.6
	Magnesium	-	-	3.0	3.0	0.8-1.0
Buffers (mM/L)	Lactate	-	28	-	-	-
	Acetate	-	-	27	27	-
	Gluconate	-	-	23	23	-
pH		4.5-7	6.5	7.4	6.6	7.35-7.45
Osmolarity (mOsm/L)		308	273	294	294	291
Strong ion difference (mM/L)		0	28	~50	~50	36-40

*NS* 0.9% sodium chloride, *LR* lactated Ringer’s solution, *PL* Plasma-Lyte A, *NoR* Normosol-R.

## Indications for balanced crystalloids

IV fluid boluses increase intravascular volume, enhance preload and improve tissue perfusion. In the 9^th^ edition of the textbook of Neonatal Resuscitation, a volume expander is indicated for infants with persistently low heart rate that does not respond to effective ventilation, chest compressions, and epinephrine associated with history of blood loss or signs of shock [1]. The recommended crystalloid solution for acute treatment of hypovolemia is NS, although LR is an acceptable alternative. The main concerns with the use of LR for neonatal resuscitation are the lack of availability in some delivery rooms and incompatibility with red blood cells [1]. Despite these limitations, some data show that balanced crystalloids provide superior resuscitative outcomes compared to NS [13–16], although most results have shown only a modest effect size and have been inconsistent.

Traditional teaching of acid-base physiology is based on Henderson-Hasselbach equation emphasizing the importance of bicarbonate. Metabolic acidosis arises when there is a decrease in blood pH and bicarbonate levels or an increase in hydrogen ions. An alternate approach to acid-base physiology was proposed by Peter Stewart in 1978 [17, 18]. According to the Stewart hypothesis, the strong ion difference (SID), the difference in concentrations between strong cations and anions, is an important factor in pH regulation. Sodium accounts for most strong cations and chloride for most strong anions, thus are major contributors of SID [19]. When chloride increases disproportionately the SID decreases, forcing bicarbonate concentration and pH downward [20]. Normal plasma has an SID of 36–40 mM/L (Table 1). Use of NS can contribute to worsening metabolic acidosis given the supraphysiologic chloride concentration and lower pH [21, 22]. Each 10 mL/kg NS bolus delivers 1.54 mM/kg of chloride. In premature infants, recommended chloride intakes vary, but cumulative intakes above 10 mM/kg from all sources in the first 3 days (3.3 mM/kg/day on average) have been associated with an increased risk of hyperchloremia [23]. Groh-Wargo and colleagues also reported that a total chloride load exceeding 6 mM/kg/day correlated with a higher incidence of metabolic acidosis in low birth weight infants [24]. Given the immature renal tubular function and impaired acid-base regulation in extremely preterm infants, this potential hyperchloremic acidosis induced by NS represents an additional physiological burden.

Balanced crystalloids have been shown to be superior for fluid bolus therapy in terms of electrolyte disturbance and acid-base balance, owing to their added buffering agents and sodium and chloride concentrations closer to physiologic levels. Arya et al. conducted a double-blind RCT that compared PL to NS as fluid bolus choice in children with shock (mean age PL group 2.81 ± 3.27 y, NS group 3.44 ± 4.01 y) and found that the PL group showed better improvement in acidosis, lower rise in serum chloride level, shorter hospital stay, lesser need for vasoactive drugs, and lower mortality [25]. Agarwal and colleagues also conducted a double-blind RCT comparing NS vs. LR as the initial fluid (used for both resuscitation and replacement) for children in diabetic ketoacidosis (median age LR group 6 y (interquartile range 3–8), NS group 7 y (interquartile range 4–9)). They observed significant hyperchloremia at 4 and 8 h in the NS group, while the rise in bicarbonate from baseline to 12 h was significantly higher in the LR group [26]. Most available studies exclude neonates, use total fluid volumes that exceed 10 mL/kg, and often evaluate chronically infused fluids rather than purely bolus therapy, which limits how well pediatric and adult data can be applied to the NICU population.

## Challenges with balanced crystalloids

There are several challenges with the use of balanced crystalloids compared to NS. A major barrier for LR relates to incompatibility issues with common medications in the NICU. The calcium in LR can interact with citrate and induce coagulation of blood products in the IV tubing [1]. Additionally, there is a risk of precipitation if LR is used concurrently with TPN, ceftriaxone, lorazepam, and phenytoin, among other medications [27]. Neither PL or NoR have calcium so do not carry the same risk of precipitation with blood products or ceftriaxone. PL and NoR have more limited data regarding compatibility with other commonly used medications but overall appears to be better than LR [28, 29].

Organ dysfunction is another challenge with the use of balanced crystalloids. Given the liver’s role in metabolizing lactate, administering LR to a patient with impaired hepatic function can lead to lactate accumulation, potentially exacerbating metabolic imbalances. The addition of potassium in these solutions may contribute to hyperkalemia, particularly in patients with an already elevated serum potassium or renal impairment. However, the potassium concentration in PL and NoR is close to human serum and thus likely has a negligible effect on overall serum potassium level, as has been shown in the literature [25, 30, 31]. Additionally, the buffering agents in balanced crystalloids may help prevent acidosis-induced potassium shifts [32]. The magnesium load in NoR is not insignificant and may preclude its use in neonates with known or suspected neonatal hypermagnesemia.

There is a common concern that LR can worsen lactic acidosis. The lactate in LR is present as sodium lactate, which is metabolized into bicarbonate and other energy substrates upon administration. Studies indicate that LR may elevate serum lactate levels but without significantly worsening acidosis [14, 33]; however, approximately 30 mL/kg of LR needs to be infused to increase serum lactic acid by 1 mM/L. Case examples of electrolyte shifts following NS and/or LR boluses in 5 different patients is provided in Table 2.

**Table 2:** Blood gas and electrolyte measurements associated with IV fluid solution and volume received.

Patient^a^	IV fluid solution	IV fluid volume (mL/kg)	Time of blood gas relative to IV bolus	pH	Base Excess/ Deficit (mEq/L)	Na (mEq/L)	K (mEq/L)	Cl (mEq/L)	LA (mM/L)
A	NS	20	Before	7.29	-8	135	4.0	106	-
			After	7.21	-11	130	3.3	106	-
B	LR	20	Before	7.37	-4	131	4.4	108	-
			After	7.34	-3	129	4.9	106	1.8
C	LR	10	Before	7.15	-6	134	4.5	106	1.3
			After	7.35	-2	136	3.8	110	-
D	NS	10	Before	7.32	-7	133	2.5	101	3.9
			After	7.29	-3	136	2.5	103	2.0
	LR	10	Before	7.08	-11	136	2.6	97	2.0
			After	7.37	-3	134	2.6	103	2.7
E	NS	10	Before	7.33	-1	130	4.8	103	-
			After	7.40	0	137	4.6	110	-
	LR	10	Before	7.33	-8	137	4.5	113	-
			After	7.36	-5	138	4.2	111	-

*NS* 0.9% sodium chloride, *LR* lactated Ringer’s solution, *LA* lactic acid.

^a^Patient A was born at 38 6/7 weeks gestation with known neural tube defect and received two 10 mL/kg NS boluses in the first 12 h of life for worsening metabolic acidosis.

Patient B was born at 38 1/7 weeks gestation with gastroschisis and received one 20 mL/kg LR bolus for base deficit on blood gas and high gastric tube output after bowel reduction and silo placement on day of life 1.

Patient C was born at 29 6/7 weeks gestation and received one 10 mL/kg LR bolus for hypotension on day of birth.

Patient D was born at 25 1/7 weeks gestation and received one 10 mL/kg LR bolus for hypotension with base deficit on blood gas on day of life 25 and one 10 mL/kg NS bolus for hypotension with base deficit on blood gas in the post-operative period status post exploratory laparotomy for small bowel volvulus requiring small bowel resection and creation of end ileostomy and mucous fistula on day of life 26.

Patient E was born at 30 2/7 weeks gestation and received one 10 mL/kg NS bolus for hypotension on day of birth and one 10 mL/kg LR bolus for hypotension with base deficit on blood gas on day of life 3.

## Practical approach for NICU patients

We propose an individualized, precision-medicine based practical approach to the use of balanced crystalloid solutions in our NICU patient population. Use of balanced crystalloids may be preferred in a neonate with hypoperfusion and known or highly suspected history of fluid loss with low preload or a neonate with existing metabolic acidosis with elevated chloride and/or low bicarbonate. Alternatively, use of NS may be preferred in situations where a neonate has limited IV access and/or requires other calcium-, phosphate-, or citrate-containing solutions or a neonate resuscitated in the delivery room when a balanced crystalloid solution is not as readily accessible. A quick reference guide is shown in Table 3.

**Table 3:** Quick reference of clinical scenarios and proposed fluid bolus type.

Clinical scenario	Preferred fluid bolus type
Intravenous access – Single site	NS
Intravenous access – Multiple sites	LR, PL, or NoR
Metabolic state – Acidosis	LR, PL, or NoR
Hypochloremia	NS
Potential need to use the same site for PRBCs or TPN	Avoid calcium-containing solutions such as LR; Preferred solution – NS; May consider PL or NoR in some cases

*NS* 0.9% sodium chloride, *LR* lactated Ringer’s solution, *PL* Plasma-Lyte A, *NoR* Normosol-R, *PRBC* packed red blood cells, *TPN* total parenteral nutrition.

## Conclusion

Metabolic acidosis is common in preterm and term newborns and optimizing IV solutions with base may help improve pH. Balanced crystalloids as the initial fluid choice show modest but mixed outcomes in children and adults. NS is associated with hyperchloremic metabolic acidosis whereas balanced crystalloids better preserve acid-base status, making them a reasonable choice in certain situations. LR use is limited by product incompatibilities, with NoR or PL as more physiologic, but costly, alternatives. Ultimately, the lack of robust evidence regarding optimal fluid type, dosing, and appropriate indications in neonates highlights the need for targeted studies, including better understanding of base sources for treating metabolic acidosis. Until such evidence is available, we described a situation-dependent, case-by-case practical approach to the use of balanced crystalloids in the NICU.
